# A heuristic model for the cost of capital of healthcare facilities: estimates for five countries

**DOI:** 10.1186/s12913-025-13451-9

**Published:** 2025-09-30

**Authors:** Sergio Zuniga-Jara, Sofia Ruiz-Campo, Karla Soria-Barreto

**Affiliations:** 1 Universidad Católica del Norte, Coquimbo, Chile; 2 Universidad Francisco de Vitoria, Madrid, Spain

**Keywords:** Healthcare facilities, Investments, Cost of capital, Weighted average cost of capital, Capital asset pricing model, Net present value

## Abstract

**Background:**

The healthcare industry has seen increasing demand, making it an attractive sector for long-term investment. Investors typically assess feasibility through an investment evaluation process that includes calculating the cost of capital and net present value. However, existing research has primarily focused on cost of capital methodologies for large corporations, often overlooking small and medium-sized enterprises. This study aimed to address that gap by proposing a simplified model to estimate the cost of capital for healthcare facilities, with particular emphasis on smaller organizations. The model incorporated key explanatory variables, including investment magnitude, organizational size, administrative expertise, and the geographical region of investment. To illustrate its applicability, we provided estimates for Chile, Germany, Spain, the United Kingdom, and the United States of America.

**Methods:**

We reviewed existing research on techniques for estimating capital costs for healthcare facilities. Building on these foundations, we developed an approach that is straightforward, adaptable, and based on publicly accessible data. Our method segmented the cost of capital into two components: (i) risks associated with the country and sector in which the investment is made, and (ii) supplementary risks arising from the investment’s scale, age, or stage of development.

**Results:**

We provided a detailed explanation of the proposed method and presented numerical estimates of the cost of capital for healthcare facilities of various sizes across several countries. The final quantitative assessments of the cost of capital ranged from 14.6% and higher, depending on the specific attributes of the investments.

**Conclusion:**

This study introduced a novel, straightforward, and user-friendly method for estimating capital costs in the healthcare sector. The model accounted for various factors influencing investment risk, including investment size, the experience level of the management team, and the country of investment. In summary, the approach supports the estimation of the cost of capital for healthcare facility investments and can be used by policymakers and healthcare leaders to make informed decisions on new investments.

## Background

Investments in new healthcare facilities are important as they lead to greater accessibility to health services, resulting in higher patient satisfaction and improved quality of life for communities [1, 2]. However, investors must allocate financial resources to pursue these investments, whether in the private or public sector. They require that the projected return on an investment be at least sufficient to counterbalance the potential return from an alternative investment of comparable risk [3, 4].

Determining the profitability of a long-term investment is complex. It involves making long-term projections of the investment’s revenues, expenses, and initial fixed investment, followed by calculating the anticipated rate of return. The healthcare sector faces additional challenges owing to the unique characteristics of different geographic regions [5].

The process of determining the profitability of a long-term investment is intricate. It entails forecasting the investment’s income and expenses over time, estimating the expected cost of capital, and ultimately calculating the net present value (NPV). The NPV is a fundamental financial instrument for assessing and selecting investments [6]. This process is particularly complex in the healthcare industry due to the distinct attributes of various regions. For example, an NPV calculation is typically required when funds are limited and a choice must be made between acquiring a CT scanner or a magnetic resonance imaging (MRI) machine [7].

To calculate the NPV, an estimate of the investment’s risk-adjusted cost of capital rate is required, as the standard NPV equation is:

$$\:NPV=\sum\:_{n=1}\frac{{FCF}_{n}}{{(1+\text{E}\left({\text{R}}_{\text{i}}\right))}^{n}}$$

where $\:E\left({R}_{i}\right)$ represents the expected cost of capital rate [6]. Therefore, a precise assessment of the cost of capital, E(R_i_), is essential for every healthcare facility investment plan [8]. An undervalued cost of capital could lead to the approval of low-return investments, while an overestimated cost could result in the rejection of promising initiatives. Typically, higher-risk investments require a higher cost of capital to ensure adequate compensation. Riskier projects significantly impact the estimated NPV, reducing the attractiveness of certain investments. Consequently, the cost of capital in emerging countries tends to be higher than in other regions due to increased uncertainty.

The methods for calculating the cost of capital are generally divided into two primary categories.

1. Traditional techniques that rely on nearly complete information, including the standard Capital Asset Pricing Model (CAPM) [9–11], its key extensions, and the so-called “accounting approach.”
2. Techniques based on incomplete information, such as methods employing CAPM and the betas of comparable companies, heuristic models, or expert surveys.

CAPM-based models estimate the expected cost of equity as the sum of the risk-free interest rate ($\:{R}_{f}$), typically measured as the yield on the United States of America (USA) government bonds, and an equity risk premium:

$$\:E\left({R}_{i}\right)=\:{R}_{f}\:+\:\left(E\left({R}_{m}\right)\:-{\:R}_{f}\right)*{\beta\:}_{i}$$

where $\:E\left({R}_{i}\right)$ is the expected return on asset i, R_f_ is the return on a risk-free asset, $\:E\left({R}_{m}\right)$ is the expected return on the market portfolio of all risky securities, and $\:{\beta\:}_{i}$ is the sensitivity of asset i to market movements, also known as the systematic risk coefficient. Several studies indicate that beta is not the only relevant measure of systematic risk [12], necessitating model extensions.

An intertemporal CAPM was proposed by Merton [13], extending the traditional $\:{\beta\:}_{i}$ by introducing a second source of risk, resulting in a two-factor model. The new risk factor, $\:E\left({R}_{n}\right)$, represents the return on an asset negatively correlated interest rate changes. In addition, $\:{\lambda\:}_{1}$and $\:{\lambda\:}_{2}$ were reformulated to consider three types of systematic risks, expressed as:

$$\begin{aligned}\:E\left({R}_{i}\right)\:&=\:{R}_{f}\:+\:\left(E\left({R}_{m}\right)\:-{\:R}_{f}\right)*{\lambda\:}_{1}\cr&\quad+\:\left(E\left({R}_{n}\right)\:-\:{R}_{f}\right)*{\lambda\:}_{2}\end{aligned}$$

Another CAPM extension is the Arbitrage Pricing Theory (APT), proposed by Ross [14]. This model introduces multiple risk factors and is expressed as:

$$\begin{aligned}\:E\left({R}_{i}\right)\:&=\:{R}_{f}\:+\:{RP}_{1}*{\beta\:}_{i1}\cr&\quad+\:{RP}_{2}*{\beta\:}_{i2}+\dots\:+{RP}_{n}*{\beta\:}_{in}\end{aligned}$$

where $\:{RP}_{i}$ is the systematic risk factor or risk premium, and “β” denotes the sensitivity of asset i to factor n, commonly referred to as “factor loading.” The APT generalizes CAPM by recognizing that, for factor 1, $\:{RP}_{1}$ is the risk premium $\:\left(E\left({R}_{m}\right)\:-\:{R}_{f}\right)$, and $\:{\beta\:}_{i1}$ is the asset’s covariance risk with market portfolio “m.” Thus, CAPM is considered a special case of the APT.

Several other significant empirical extensions to the CAPM have also been proposed, including the three-factor model by Fama & French [15], the “Momentum” factor by Jegadeesh & Titman [16], the higher-moment CAPM examined by Doan et al. [17], the international CAPM (I-CAPM) proposed by Dumas & Solnik [18], and the five-factor model by Fama & French [19]. In addition, various risk premiums have been incorporated into the traditional CAPM, such as the “Small Firm Premium,” “Illiquidity Premium,” “Country Risk Premium,” and “Company-Specific Risk Premium.”

Finally, heuristic methods encompass a set of approaches used when no clear algorithmic solution exists. These methods rely on experience, creativity, and lateral thinking to identify workable solutions.

### How current models are insufficient in addressing the issue

The aforementioned models often rely on data from publicly traded enterprises—companies with shares listed on various stock markets. However, the minimal required rates of return for small businesses are significantly higher. Table 1 presents an overview of multiple studies reporting return rates for such companies in the USA, typically ranging within 30–55%. These values are considerably higher than the average returns of publicly traded firms, which generally range within 10–15%—the long-term average return of the S&P 500. Table 1, adapted from Zuniga-Jara & Soria-Barreto [20], supports the notion that small enterprises have substantial risk premiums, which should be incorporated when calculating their cost of capital. These returns may seem high at first glance; however, they can be better understood through the following analogy: if an entrepreneur demands a 50% return on their equity (not their debt), this simply implies that they expect a two-year payback period on their own funds. This is quite realistic in the context of small, high-risk ventures.

**Table 1:** Required returns on equity for small businesses (SMEs) in the united States of America

Authors	Cost of equity for SMEs
Wetzel [21]	50%
Ruhnka & Young [22]	54.80%
Plummer [23]	40.6%-59.6%
Gompers and Lerner [24]	30.50%
Guenther and Willenborg [25]	29%
Bygrave, Hay, and Peeters [26]	50%
Kerins, Smith, and Smith [27]	45.60%
Manigart et al. [28], using category 6	46%-55%
Conrick [29], using EBIT	49.90%

### Estimates of the cost of capital in the healthcare sector

Various researchers have conducted empirical studies to estimate the global cost of capital in the healthcare sector. Table 2 indicates that the CAPM is the most commonly employed method for these estimations.

**Table 2:** Research involving capital cost estimates in the healthcare industry

Authors	Period & Country	Sector	Cost of Capital (%)	Model	Source of Financial Data
Sloan et al. [4]	1972–1988, USA	Non-profit hospitals	13.2	CAPM and APT	NYSE
		For-profit hospitals	18.5		
Harrington [2]	2006–2008, USA.	Pharmaceutical	9.3/9.1	CAPM and Fama & French [15]	S&P 1500
		Biotechnology	11.8/12.9		
		Health devices	11.2/13.7		
Giaccotto et al. [30]	1950–2004, USA.	Pharmaceutical	2.8	CAPM	Center for Research in Securities Prices (CRSP)
Vecchi et al. [31]	1997–2011, U.K.	Investments	6.38/17.62	CAPM	U.K. government bonds; FTSE U.K. Index; Bloomberg
Vernon et al. [32]	1980–2006, USA.	Pharmaceutical	10.39	CAPM	S&P 1500, CRSP
			16.61	Fama & French [15]	
Kerr et al. [33]	2007–2011, Australia	Hospitals	9.0/9.4	CAPM	Productivity Commission, Australian Institute of Health and Welfare
Kossova & Sheluntcova [34]	2000–2012, Russia	Healthcare facilities	10.4	CAPM	Federal State Statistics Service of Russia
		Hospitals	9.0		
Hellowell [35]	2012, UK	Hospitals	15.0	CAPM	Markets data section of the *Financial Times*
Moro [36]	2009–2012, Italy	Investments	7.87	CAPM	Investment simulations
Holcomb & Smith [37]	2020, USA.	Hospitals	Negative	CAPM	S&P 500 Index

Source: Prepared by the authors

All estimations in Table 2 were based on stock market data and, consequently, applied only to large, publicly listed corporations. As shown, the methodologies used in these studies did not sufficiently estimate the cost of capital for small or early-stage firms in the healthcare sector. The table also shows that non-profit hospitals usually require lower returns than for-profit hospitals (Sloan et al. [4]), which is because the cost of equity capital is typically used in the former case. The model proposed in this study focuses on the latter type of institution.

This study addresses that gap by proposing a methodological framework for calculating the cost of capital in the healthcare industry, applicable to investments of any size—whether by large corporations or smaller entities. The model incorporates key explanatory variables, including investment size, company scale, managerial expertise, and geographic location. A numerical example is provided for Chile, Germany, Spain, the United Kingdom, and the USA.

The article is structured into four sections. The second section outlines the research methodology employed in this study. Section “Results” presents the research findings and their relevance to the healthcare industry. Section “Discussion” contains the discussion. The final section presents the conclusions, implications and limitations.

## Methods

This section outlines the heuristic approach employed to estimate the cost of capital for small healthcare facilities. The proposed model defines the cost of capital, $\:Rz$, as the sum of two risk premiums ($\:{RP}_{x,y}$ and $\:{RP}_{z}$) and the risk-free rate ($\:{RF}_{\left(x\right)})$, expressed as:

1 $$\:{R}_{z}={RF}_{\left(x\right)}+{RP}_{x,y}\:+\:{RP}_{z}$$

where

- $\:{RF}_{\left(x\right)}$ is the risk-free rate of country “x”.
- $\:{RP}_{x,y}$ is the risk premium associated with the specific country (y) and industry (x) in which the investment has been made. Although this model is applied to the healthcare sector, it is adaptable to other industries.
- $\:{RP}_{z}$ incorporates the additional risk premiums related to the investment’s size, stage of development, or age. For publicly traded large enterprises in the USA, $\:{RP}_{z}$ = 0. However, for unlisted, small, or early-stage firms, $\:{RP}_{z}$ >0.

The following sections provide a detailed review of both risk premiums.

### $\:{\varvec{R}\varvec{P}}_{\varvec{x},\varvec{y}}$: country risk (x) and industry risk (y)

When assessing investment risks, both the country where the investment is made and the specific industrial sector must be considered. The Industry Classification Benchmark (ICB) is a classification system comprising ten industries, which is further divided into 19 supersectors, 41 sectors, and 114 subsectors. The risk premium $\:{RP}_{x,y}$ for a specific industry “y”’ operating in country “x” is determined using the following formula:

2 $$\:{RP}_{x,y}=\:({RM}_{\left(x\right)}\:-\:{RF}_{\left(x\right)})*{Beta}_{(US,y)}$$

This formula follows the traditional CAPM specification of the risk premium, where $\:{RM}_{\left(x\right)}$ represents the market return of country “x.” The $\:{Beta}_{(US,y)}$ refers to the mean systematic risk of the publicly traded companies in country “x”’ within the industrial sector “y.” The rationale for adopting estimates from the USA $\:{Beta}_{(US,y)}$ as a substitute for the Beta coefficient of any country “x” is based on the assumption that the most reliable estimates of an industry’s inherent risk can be derived from the industry Beta in the USA. The major USA stock exchanges benefit from extensive global integration, characterized by high liquidity and numerous publicly traded companies. Moreover, the USA market provides comprehensive publicly available information across various industrial sectors, thereby rendering it a reliable source for industry risk assessments. Several researchers have also contributed to the estimation of systematic risk for USA industries. Among them, Vyas and van Baren [38] and Damodaran [39] recently reported freely available beta estimates for different sectors in the USA.

To calculate RF_(x)_ for each country “x,” we combined the “Country Default Spread (CDS)” of that country with the risk-free rate of the USA. The USA risk-free rate is typically determined by the yield on 10-year Treasury bonds. This is expressed as:

$$\begin{aligned}\:{RF}_{\left(x\right)}&=\:{RF}_{US\:10-year\:T-Bond}\cr&\quad+\:{Default\:Spread}_{\left(x\right)}\end{aligned}$$

To estimate the market return for each country, $\:{RM}_{\left(x\right)}$, the methods proposed by Qin & Pattanaik [40] and Erb et al. [41] were adopted. They utilized the “Country Credit Rating Model (CCR Model),” which relies on sovereign credit ratings. The equation used is:

$$\:{RM}_{\left(x\right)}\:=\:\mu\:0\:+\:\mu\:1*ln\left(CCR\right(x\left)\right)$$

where $\:{RM}_{\left(x\right)}$ is the market return for country “x,” CCR is the country’s credit rating, and µ_0_ and µ_1_ are empirically parameters over an extended period.

### $\:{\varvec{R}\varvec{P}}_{\varvec{z}}$: size and development stage risk based on a heuristic approach

The risk premium $\:{RP}_{z}$ compensates for size and development stage risks. Based on Eq. 1, $\:{RP}_{z}$ is a risk premium related only to size and state of development of the firm, something that is not captured directly by $\:{RP}_{x,y}$. Consequently, it is defined as:

3 $$\:{RP}_{z}\:=\:\left({RM}_{z}-{RF}_{\left(US\right)}\right)*{Beta}_{z}$$

where $\:{RM}_{z}$ is the expected return on a portfolio of non-publicly traded companies, with an average sensitivity of 1 to the macroeconomic factor associated with “size and development stage” and a sensitivity of 0 to remaining risk factors. Further, $\:{Beta}_{z}$ indicates the effect of risks on the performance of an investment or firm. For publicly traded firms, $\:{Beta}_{z}$ = 0, whereas for non-publicly traded, small, or early-stage firms, $\:{Beta}_{z}$ >0.

Our primary objective is to obtain estimates of $\:{Beta}_{z}$ in Eq. 3, to then determine $\:{R}_{z}$ in Eq. 1 for companies having different size and development risks. Instead, the term $\:\left({RM}_{z}-{RF}_{\left(US\right)}\right)$ remains constant for any risk category.

The empirical estimation methodology to obtain the components of $\:{RP}_{z}$ (Eq. 3) is as follows. Limited information is available on investor-required returns for various stages of venture development ($\:{R}_{z}$). However, certain estimates of $\:{R}_{z}$ exist for several categories of small, privately held firms. Based on these, $\:{RM}_{z}$ is obtained by averaging the investor-required returns for several available risk categories. $\:{RF}_{\left(US\right)}$ is simply the USA risk-free rate, as the source of the $\:{R}_{z}$ estimates is from entrepreneurs in the USA.

To estimate $\:{Beta}_{z}$ for several categories of companies, the process employed is as follows. We have estimates of $\:{R}_{z}$ for each category. Consequently, based on the estimates of $\:{RF}_{\left(US\right)}$ and $\:{RP}_{x,y}$ in Eq. 1, through subtraction, we estimate an $\:{RP}_{z}$ for each category. However, $\:{RP}_{x,y}$ depends on the value of $\:{Beta}_{\left(US,y\right)}$, and the available estimates of $\:{R}_{z}$ do not tell provide insights on the economic sector (they originate from the USA). Thus, our best prediction is that $\:{Beta}_{\left(US,y\right)}$ has a value of 1.0, that is, an average systematic risk for the industry. Consequently, we obtain an $\:{RP}_{z}$ for each category, and we attain all the other parameters (except $\:{Beta}_{z}$). The estimated values ​​of $\:{RM}_{z}$ and $\:{RF}_{\left(US\right)}$ are used to calculate the value of $\:{Beta}_{z}$ as $\:{RP}_{z}$ divided by the difference of $\:{RM}_{z}$ (non-publicly traded) and $\:{RF}_{\left(US\right)}$. This calculation yields a $\:{Beta}_{z}$ value for each business category. The estimation of these coefficients provide a measure of risk for each category and can be extrapolated using existing data about the firm or investment under consideration.

Thus, the model of Eq. 1 can be expanded as follows:

4 $$\begin{aligned}\:{R}_{z}\:&={RF}_{\left(x\right)}+\left[({RM}_{\left(x\right)}-{RF}_{\left(x\right)})*{Beta}_{(US,y)}\right]\cr&\quad+\left[({RM}_{z\:}-{RF}_{\left(US\right)})*{Beta}_{z}\right]\end{aligned}$$

In summary, Eq. 4 follows the structure of traditional financial equilibrium models in finance, such as the CAPM and its derivatives, where parameters are typically estimated using aggregated data from groups of firms within a sector assumed to be sufficiently homogeneous. The contribution of this model lies in extending the CAPM by incorporating country-specific, industry-specific, and firm development-stage information, thereby providing more precise and useful insights for both investors and companies. Due to data availability constraints, the model relies on U.S. information for four parameters: $\:{Beta}_{(US,y)}$, $\:{RM}_{z\:}$, $\:{RF}_{\left(US\right)}$, and $\:{Beta}_{z}$. For systematic risk in listed firms within sector y ($\:{Beta}_{(US,y)}$), the U.S. market is the only one with a sufficiently large number of listed companies to produce statistically significant industry-level estimates. Similarly, for the expected return of non-listed firms’ portfolios ($\:{RM}_{z\:}$) and for the risk associated with firm size and development stage ($\:{Beta}_{z}$), only U.S. data offer enough reliable estimates for small firms. For the same reason, the U.S. risk-free rate ($\:{RF}_{\left(US\right)}$) is adopted in the model.

We should emphasize that our focus is primarily on firms that are not publicly listed, for which existing models offer poor solutions. Our approach provides a more accurate response by incorporating country-specific, industry, and firm-level characteristics such as size and stage of development. Since small firms are not publicly traded and therefore lack firm-specific Beta estimates, we use the the industry Beta estimation as a proxy, which is available. Furthermore, when estimating the discount rate for evaluating the attractiveness of an investment—specifically for calculating Net Present Value (NPV) —, it needs to be calculated the Weighted Average Cost of Capital (WACC), not just the cost of equity ($\:{R}_{z}$). The WACC reflects company-specific characteristics, as it combines the cost of equity ($\:{R}_{z}$) weighted by the firm’s equity share in its capital structure, along with the company’s actual cost of debt, weighted by its leverage. Finally, while it might seem that the model assumes the same risk across countries within a given sector (e.g., healthcare), the formulation $\:{RF}_{\left(x\right)}+\left[({RM}_{\left(x\right)}-{RF}_{\left(x\right)})*{Beta}_{(US,y)}\right]$ explicitly incorporates country-specific variables (x), thereby capturing local risk differences.

The subsequent section presents the quantitative outcomes obtained by applying the proposed model for calculating the cost of capital ($\:{R}_{z}$). This approach is broadly applicable and can be used to assess investments in any firm, irrespective of its country, industry, size, or stage of development, as well as whether publicly traded or privately held.

## Results

The following numerical results by applying the proposed model.

### $\:{\varvec{R}\varvec{P}}_{\varvec{x},\varvec{y}}$: country risk (x) and industry risk (y)

Table 3 presents the parameter estimates of Eq. 2 for certain countries and industrial sectors. These can be used to obtain the risk premiums $\:{RP}_{x,y}$. For example, for the USA and Utility (General) sector, it is (13.03–4.10%)*0.41 = 3.66%; for Spain and Construction Supplies, it is (17.38–5.81%)*1.0 = 11.57%; for Hospitals and Healthcare, in Chile it is (22.61–5.01%)*1.17 = 20.59%, and Mexico for Healthcare (Information and Technology), it is (29.25–6.14%)*1.47 = 33.98%. Similarly, estimates of $\:{RP}_{x,y}$ can be obtained for any country “x” and any sector “y” by replacing that country’s data in $\:{RF}_{\left(x\right)}$ and $\:{RM}_{\left(x\right)}$ (the data for $\:{Beta}_{(US,y)}$ will be those of the USA, as explained in the methodology).

**Table 3:** Estimates of $\:{RM}_{\left(x\right)}$, $\:{RF}_{\left(x\right)}$, and $\:{RP}_{x,y}$ for certain sectors in selected countries

Country	Credit Rating Rate (CCR) (1)	RM_(x)_ (2)	Country Default Spread (CDS) (3)	RF_(USA)_ (4)	RF_(x)_ (3)+(4)	Sector	Beta(US, y) (5)
Brazil	34.9	33.03%	3.22%	4.10%	7.32%	Utility (General)	0.41
Chile	57.4	22.61%	0.91%	4.10%	5.01%	Construction Supplies	1.00
Germany	90.9	12.99%	0.00%	4.10%	4.10%	Hospitals and Healthcare	1.17
Mexico	41.8	29.25%	2.04%	4.10%	6.14%	Healthcare (Inf. and Tech.)	1.47
Spain	73.7	17.38%	1.71%	4.10%	5.81%		
United Kingdom	87.8	13.71%	0.64%	4.10%	4.74%		
United States of America	90.7	13.03%	0.00%	4.10%	4.10%		

(1) Country data sourced from reference [41], Exhibit 6

(2) Values for RM(x) were derived using an intercept of 53.71 and a slope of -10.47 in reference [41]

(3) Country Default Spread (CDS) values obtained from reference [42]

(4) USA Treasury 10-year bond rate retrieved from the Federal Reserve of St. Louis (FRED)

(5) Beta coefficients based on data from Damodaran [39] for the “Average Levered Beta.”

### $\:{\varvec{R}\varvec{P}}_{\varvec{z}}$: size and development stage risk based on a heuristic approach

Most existing data on $\:{R}_{z}$ for small and newly established enterprises have been derived from surveys of investors and entrepreneurs in the USA. Estimates of $\:{R}_{z}$ for different business life stages have been provided by [42]. The riskiest enterprises—those categorized as “Seed-stage”—have required returns significantly high, ranging from 73% [22] and 50% [21] to 75.4% and 49.2% [23]. However, these characteristics typically comprise microenterprises run by only one or two individuals with limited prior experience.

Table 4 presents the mean return estimates from Ruhnka and Young [42], organized according to the heuristic approach proposed by [43, 44] for determining the cost of capital. The average $\:{R}_{z}$ values for each of the six categories are 70%, 52%, 41%, 35%, and 32%, and the final category includes large publicly traded firms, exhibiting an average capital cost of 13%. Note that the ranges in Table 4 require some flexibility. For example, a hospital with 300 employees that is not publicly traded should likely be considered under the Exit Stage category (Going Public). Likewise, listed chains such as HCA, with a market capitalization exceeding USD 90 billion, or Fresenius, which operates large networks in Germany and Spain, should properly fall under the Share Company category in the table. All estimates are calculated based on data from the USA, which is not a limitation in this context because the country risk component is captured separately in the other component of the model ($\:{RP}_{x,y}$). Thus, to estimate $\:{RP}_{z}$, which only contains the risk associated with size, we data from any country can be used, provided the estimates are available and sufficiently reliable. Therefore, to estimate the market return of the last component of Eq. 4, that is, for non-publicly traded assets, average of the five $\:{R}_{z}$ values was used, yielding $\:{RM}_{z\:}$=46%.

Let us focus on Eq. 4 now. As the five data points for $\:{R}_{z}$ data originate from firms in the USA, they are used to calibrate the model. We take $\:{RF}_{\left(x\right)}$= 4.1%; and $\:\left[({RM}_{\left(x\right)}-{RF}_{\left(x\right)})*{Beta}_{(US,y)}\right]$= 8.93% as the estimates of the first two terms of Eq. 4. By difference, we estimate $\:{RP}_{z}=\left[({RM}_{z\:}-{RF}_{\left(US\right)})*{Beta}_{z}\right]$; each category yields values of 57%, 39%, 28.0%, 22.0%, 19.0%, and 0.0%. The last category includes results for large, listed companies with $\:{RP}_{z}$=0. Notably, the value of $\:{RF}_{\left(x\right)}$ and $\:{RP}_{x,y}$ in this calibration are the same for all categories. This is because belonging to a category only affects the value of the third component, $\:{RP}_{z}$, in Eq. 4. The column headings of Table 4 state that these are the average estimates. For example, for Category 3 (Third stage), the calculation is 28%/(46%−4.1%) = 0.67. Moreover, $\:{Beta}_{z}$ (which equaled zero for publicly traded firms) increased as the organization or project belonged to an earlier stage of development.

**Table 4:** Mean returns and$\:{Beta}_{z}$according to size and stage of development

Category or Stage	The life cycle and size of the firm that would undertake the investment being assessed	*R*_z_ Average	RP_z_ Average	$\:{Beta}_{z}$
First Stage (seed)	Microenterprises with 1 or 2 people, operating for less than two years. In the USA, investors typically expect mean returns of approximately 70%.	70.0%	57.0%	1.36
Start-up (Second Stage)	Microenterprises that depend on the specialized skills of one or two people, with fewer than 10 employees and often managed by a single professional. In the USA, investors typically expect mean returns of approximately 52%.	52.0%	39.0%	0.93
Third Stage (Small Business)	Small businesses with 11–49 employees, often family-run. In the USA, investors typically expect mean returns of approximately 41%.	41.0%	28.0%	0.67
Fourth Stage (Venture Capital)	Medium-sized companies with 50–250 professionals. In the USA, investors typically expect mean returns of approximately 35%.	35.0%	22.0%	0.53
Exit Stage (Going Public)	Medium-sized companies with the potential to go public. These firms have stable past earnings and fairly predictable futures. In the USA, investors typically expect mean returns of approximately 32%.	32.0%	19.0%	0.45
Share Company in US	Large companies with over 250 employees. Their financing is partially public, and financial information is available to the public. The total return of these companies in a given country represents the market return (Rm). This category includes established firms with a strong market position, stable past earnings, and predictable futures. In the USA, investors typically expect mean returns of approximately 12%.	13.0%	0.0%	0.00

Source: Based on Table 2 and data from [42–44]

## Cost of capital for healthcare facilities: an illustration for five countries

To illustrate the method, we estimated the cost of capital for healthcare facilities in five countries: Chile, Germany, Spain, the United Kingdom, and the USA. These estimates were calculated for each of the five primary categories of organizations shown in Table 4.

Table 3 presents the values of market return $\:{RM}_{\left(x\right)}$ and the risk-free rate $\:{RF}_{\left(x\right)}$ for these countries. To estimate the Beta risk coefficient ($\:{Beta}_{(US,\:y)}$) for the healthcare sector, we used the “Average Levered Beta” from the “Hospitals/Healthcare Facilities” sector, $\:{Beta}_{(US,y\:=\:Hospitals/Healthcare\:Facilities)}$ = 1.17. In addition, the country risk premium and industry sector risk ($\:{RP}_{x,y}$) were calculated accordingly.

**Fig. 1:**
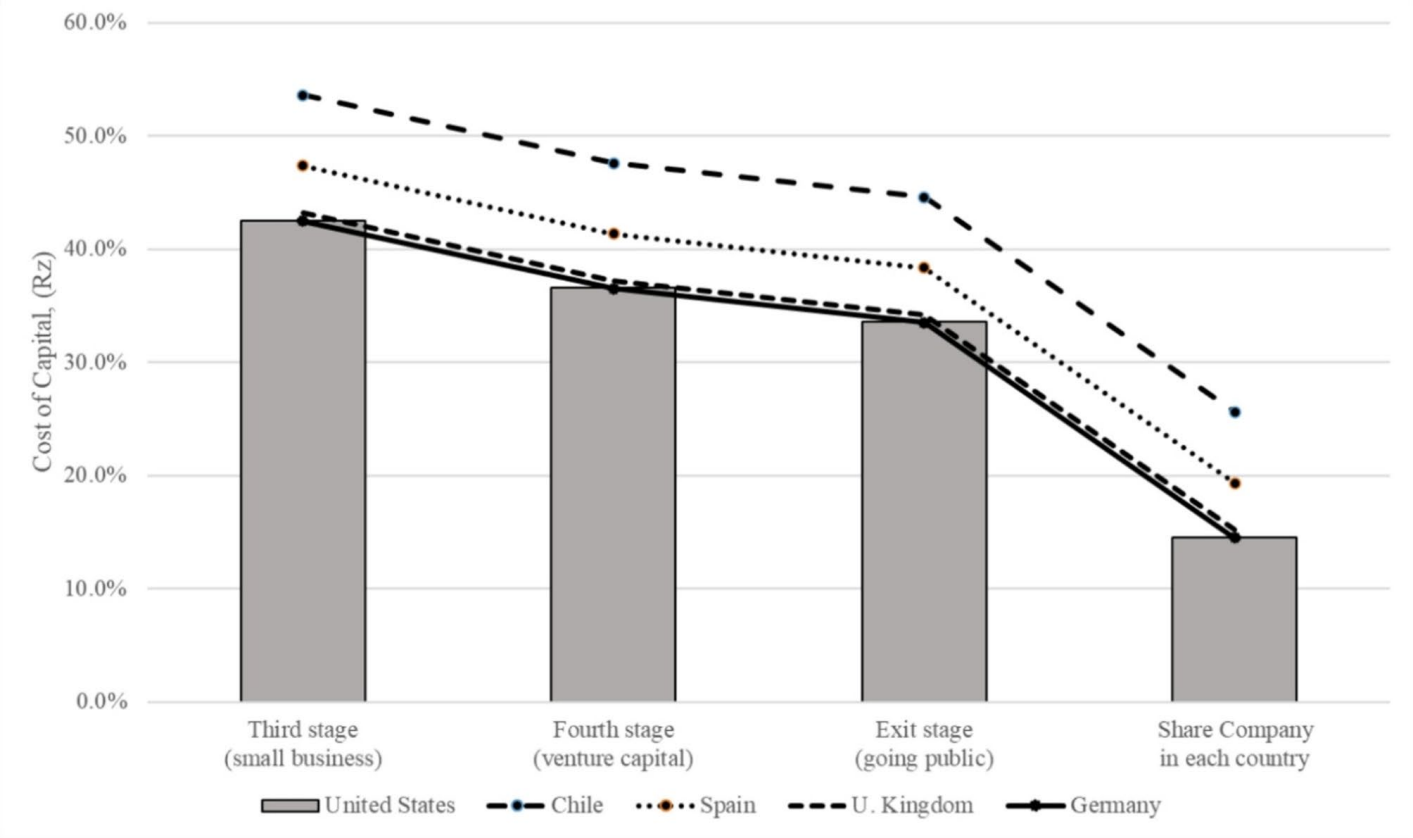
Cost of capital estimates for healthcare facility investments in different countries

Table 5 presents the final results of the proposed methodology for calculating the cost of capital in the healthcare facilities industry across five countries. We found that $\:{RP}_{x,y}$ remained constant with each category. Variations occurred only owing to differences in country risk or industry sector risk. In addition, the structure of $\:{RP}_{z}$ remained uniform across all countries, changing only when transitioning from one category to another. Table 5 further indicates that the $\:{RP}_{z}$ value was consistently zero for large publicly listed companies in both the USA and other countries. Therefore, $\:{R}_{z}$ in Fig. 1 can be extrapolated universally to organizations of any size, sector, or country, with appropriate modifications.

**Table 5:** Estimates of the cost of capital for investments in healthcare facilities across selected countries

Country	Components	Third Stage (Small Business)	Fourth stage (Venture Capital)	Exit Stage (Going Public)	Share Company in Each country
Chile	RP_x, y_	20.59%	20.59%	20.59%	20.59%
	RP_z_	28.00%	22.00%	19.00%	0.00%
	R_z_	53.60%	47.60%	44.60%	25.60%
	CI 95%	(52.98%, 54.21%)	(47.03%, 48.16%)	(44.06%, 45.16%)	(25.16%, 26.07%)
Germany	RP_x, y_	10.40%	10.40%	10.40%	10.40%
	RP_z_	28.00%	22.00%	19.00%	0.00%
	R_z_	42.50%	36.50%	33.50%	14.50%
	CI 95%	(41.92%, 43.01%)	(36.01%, 36.96%)	(32.99%, 33.87%)	(14.14%, 14.84%)
Spain	RP_x, y_	13.53%	13.53%	13.53%	13.53%
	RP_z_	28.00%	22.00%	19.00%	0.00%
	R_z_	47.34%	41.34%	38.34%	19.34%
	CI 95%	(46.80%, 47.99%)	(40.84%, 41.86%)	(37.85%, 38.83%)	(18.91%, 19.69%)
United Kingdom	RP_x, y_	10.50%	10.50%	10.50%	10.50%
	RP_z_	28.00%	22.00%	19.00%	0.00%
	R_z_	43.24%	37.24%	34.24%	15.24%
	CI 95%	(46.80%, 47.99%)	(40.84%, 41.86%)	(37.85%, 38.83%)	(18.91%, 19.69%)
United States of America	RP_x, y_	10.45%	10.45%	10.45%	10.45%
	RP_z_	28.00%	22.00%	19.00%	0.00%
	R_z_	42.55%	36.55%	33.55%	14.55%
	CI 95%	(42.02%, 43.13%)	(36.04%, 36.99%)	(33.11%, 34.03%)	(14.22%, 14.92%)

CI = Confidence intervals

Upon reviewing Table 5; Fig. 1, we determined that if we assume the sector y = healthcare to be the same in all cases, the differences in return $\:{R}_{z}$ were attributable to the differences in x = country and those in the size or stage of development of the companies. In this case, it was observed that all the returns required by investors systematically decrease with the movement of the characteristics of the investments from Stage 3 (higher risk) to Stage 1 (lower risk). Furthermore, the returns also follow a fairly homogeneous pattern when changing countries, from a globally less risky country like the USA to one with higher country risk like Chile. Thus, the figure enable us to appreciate how the two different components of risk ultimately determine the return required by investors, assuming, in this case, that the industrial sector is the same (Healthcare).

The proposed methodology allowed us to estimate the cost of capital for investments that did not fit clearly align with predefined categories by size and development stage. For example, when evaluating a healthcare investment by a firm falling between Category 3 and Category 4, we interpolated $\:{Beta}_{z}$ to be 0.6. This estimation was derived by interpolating the values of both categories. In this scenario, if the healthcare investment was located in Chile, we determined the cost of capital using the following formula in Eq. 4, and substituting the values; value of $\:{R}_{z}$=5.01% + (22.6% − 5.01%)*1.17 + (46% − 4.1%)*0.6 = 50.73% was obtained. This represented the calculated cost of equity capital rate obtain through our methodology for an investment with the specified characteristics.

A Monte Carlo analysis was performed to estimate the confidence intervals shown in Table 5. The analysis used XLRISK, which is an Excel add-in [46]. In Eq. 4, for the risk-free rate ($$\:{RF}_{\left(x\right)}$$), a normal distribution with a standard deviation of 5% from the mean (5% up and 5% down) was used [47]. For the Market Return ($$\:{RM}_{\left(x\right)}$$), a normal distribution with a standard deviation of 22% from the mean was used [48]. Further, for the coefficients $$\:{Beta}_{(US,y)}$$, a normal distribution with a standard deviation of 25% of the mean was used [49]. Finally, for the heuristic component owing to $$\:{Beta}_{z}$$, a triangular distribution with a range of +/-0.25 points with respect to the central value was used. Following the iterations, the margin of error was calculated for a 95% confidence interval, as $$\:MoE=z*\left(\raisebox{1ex}{$\sigma\:$}\!\left/\:\!\raisebox{-1ex}{$\sqrt{n}$}\right.\right)$$; where z is the z-score corresponding to the desired confidence level, σ is the standard deviation of the simulation outcomes, and n = number of simulations. Consequently, using the CONFIDENCE.T function, with *n* = 1000 iterations, the confidence intervals were obtained (Table 5).

## Discussion

Investments in healthcare are crucial for meeting society’s evolving medical needs, fostering innovation, and delivering consistent financial returns, all while supporting broader socio-economic development [50]. The sector is marked by rising demand and rapid technological advancements, offering relatively stable revenues that make it attractive for long-term investment. Demographic trends, particularly the aging population in many countries, have intensified healthcare demands, further expanding investment opportunities. These dynamics enhance the sector’s appeal to investors.

When evaluating investment viability, lenders typically undertake a rigorous assessment process. This includes forecasting future costs and revenues, estimating the cost of capital, and calculating the NPV [6]. Although the literature offers several approaches for estimating the cost of capital [9, 12, 13], these are mainly tailored to large, publicly traded companies with accessible financial data. Consequently, practical methods suitable for small and medium-sized enterprises (SMEs) remain limited. To address this gap, we developed a methodology for estimating the cost of capital across investment scales—small, medium, and large.

We employed a heuristic approach to streamline the estimation process, incorporating two key risk factors: (i) a specific risk premium based on the investment’s size, age, or development stage, and (ii) national risk. Using this method, we estimated the cost of capital ($\:{R}_{z}$) for healthcare facilities in five countries. The results ranged from 14.55% for large firms in the USA (Table 5), with higher values depending on investment stage, scale, and location.

Our estimates revealed substantial variation in capital costs. In extreme cases, small-scale healthcare investments by SMEs in high-risk countries exceeded 53% (Table 5). While these figures may appear unexpectedly high, Table 1 showed that even small investments in low-risk countries like the USA could incur capital costs up to 60% [23]. For instance, investments in Stage 3 (small businesses) in developed countries such as Spain reached 47.4%, with higher figures observed in developing nations. These elevated rates can be attributed to the high failure rates of new enterprises. Previous studies report that over 80% of startups fail within their first year [51–53], with 20–40% closing within two years and only 40–50% remaining operational after seven years [54, 55]. Thus, the elevated risk of bankruptcy associated with new and small investments justifies the high cost of capital observed in the healthcare sector.

Notably, several parameters from the USA financial market were used, such as certain beta coefficients. This is based on the structural conditions that render this market among the most developed and efficient globally. In particular, it has high liquidity, depth, and low transaction costs, which facilitate the rapid incorporation of information into asset prices [56]. These characteristics favor compliance with the assumptions of financial models (such as the CAPM), particularly regarding market efficiency and the rationality of economic agents.

Our analysis primarily targeted the healthcare sector’s cost of capital, but the model, as noted earlier, is easily adaptable to other industries across different economies. However, future studies may identify additional factors that are equally or even more significant than those considered in this study. As a recommendation, this research paves the way for further investigations targeting the validation of the estimates through surveys of entrepreneurs operating at different investment scales and across various countries, considering the scarcity of publicly available data for most small enterprises.

Thus, this study provides valuable insights for potential investors in the healthcare sector, regardless of facility size, by facilitating the determination of the optimal discount rate for NPV calculations. This facilitates a more effective evaluation of the investment feasibility. This study also presents numerical illustrations of appropriate discount rates based on the proposed methodology. Accurate discount rate estimates aid investors in avoiding the allocation of capital to unviable projects and instead directing their resources toward offering the most advantageous opportunities.

## Conclusion

This study introduced a heuristic model for estimating the cost of capital in healthcare facilities, addressing a critical gap in existing methodologies that primarily focus on large corporations. By incorporating factors such as investment size, organizational scale, managerial expertise, and geographical location, we developed a clear and adaptable framework tailored to small and medium-sized enterprises in the healthcare sector.

Our approach divided the cost of capital into two main components: risks related to the country and industry, and additional risks arising from the investment’s size, development stage, or age. We presented numerical estimates for five countries—Chile, Germany, Spain, the United Kingdom, and the United States—demonstrating the model’s applicability across diverse contexts. Results indicated cost of capital rates beginning at 14.6%, with variation based on specific investment characteristics.

The heuristic model simplified the estimation process by leveraging publicly available data, making it accessible to investors in smaller healthcare organizations who may lack extensive financial expertise. By enhancing the accuracy of cost of capital calculations, the model supports more informed investment decisions in a sector crucial to improving community health outcomes.

Despite its accessibility and user-friendliness, the model has certain limitations. The study used U.S. industry beta values as proxies for systematic risk in other countries, assuming these were globally representative. However, this assumption may not fully reflect country-specific market conditions, especially in less integrated or emerging economies. Additionally, the model relied on static parameters—such as country credit ratings and industry betas—which may change over time due to economic shifts or market disruptions, potentially affecting the reliability of long-term investment decisions based on the model.

The model also simplified risk premiums into two categories: country/industry risk and size/development stage risk. While this made the tool more approachable, it may have excluded other influential factors, such as regulatory environments, political stability, or sector-specific volatility.

The study focused on five countries—Chile, Germany, Spain, the United Kingdom, and the United States—limiting the generalizability of the findings to regions with different economic conditions or healthcare systems. Nevertheless, the framework offers a practical starting point for policymakers and healthcare leaders considering new investments. Future research should broaden the model’s geographic scope, incorporate dynamic variables to reflect evolving market conditions, and validate its effectiveness through empirical applications. These developments would further refine the methodology and strengthen its relevance to global healthcare investment planning.

This study, while not involving human subjects, highlights ethical issues in healthcare investment. Cost of capital calculations should not justify underinvestment in high-risk areas. National parameters may misrepresent local needs, making transparency essential. Simplifying risk requires caution, as ethical decisions demand more than just financial data.

## Data Availability

No datasets were generated or analysed during the current study.
